# Analysis of blood donor deferral pattern in Uttar Pradesh, India

**DOI:** 10.6026/973206300211942

**Published:** 2025-07-31

**Authors:** Arvind Kumar Singh, Jyoti Kala Bharati, Aaditya Shivhare, Yatendra Mohan, Nouratan Singh

**Affiliations:** 1 Department of Transfusion Medicine, Uttar Pradesh University of Medical Sciences, Saifai, Etawah, Uttar Pradesh-206130, India

**Keywords:** Blood donation, whole blood donor, donor deferral, haemoglobin, hypertension, temporary deferral

## Abstract

Blood donor deferral is essential for safety. However, it impacts donor recruitment. Therefore, it is of interest to analyze deferral patterns at a rural tertiary care hospital in North India (March 2018-March 2023) among 45,067 registered donors. 8,159 were deferred 76.4% were temporary (low hemoglobin, recent medications) and 23.6% were permanent (hypertension, diabetes). Notably, 25% of male deferrals were due to recent alcohol intake. Hence, targeted strategies addressing these causes could improve donor retention and blood supply stability.

## Background:

Each year, blood transfusions save millions of lives worldwide, yet access to safe and timely blood remains a challenge, particularly in developing countries [1]. The availability of blood and blood products is often insufficient to meet demand, creating a significant disparity between high- and low-income regions. According to the World Health Organization (WHO), over 81 million units of blood are collected annually, but only 39% come from low-income countries, despite these nations comprising 82% of the global population [1]. Ensuring a safe transfusion system requires both scientific and technological advancements in blood processing and rigorous donor selection criteria and understanding the reasons behind donor deferrals is vital for optimizing blood donation processes [2]. Deferrals can have a negative psychological impact on donors, discouraging future participation and hindering donor retention efforts [3]. While necessary for transfusion safety, deferral policies must be balanced to maintain an adequate donor pool [4]. The donor selection process involves a thorough assessment of medical history, physical examination findings, hemoglobin levels, vital signs, and high-risk behaviours [4, 6]. The "donor questionnaire" serves as a key tool for screening donors, ensuring that those at high risk for infections or adverse donation reactions are identified and deferred appropriately [5]. Studies conducted in India have identified various common causes of donor deferral, highlighting demographic variations across different regions. Therefore, it is of interest to systematically evaluate the incidence and reasons for deferrals in a tertiary care hospital-based blood centre in North India which catering mainly rural population.

## Materials and Methods:

## Study design:

This study was a cross-sectional retrospective analysis of voluntary non-remunerated and replacement blood donors who presented for blood donation at the Blood Centre, Department of Transfusion Medicine, Uttar Pradesh University of Medical Sciences (UPUMS), Saifai, Etawah, Uttar Pradesh.

## Study population:

All blood donors were selected following the guidelines set by the Drugs and Cosmetics Act and the Directorate General of Health Services, Ministry of Health and Family Welfare, Government of India. Donor eligibility was assessed based on predefined selection criteria.

## Ethical approval:

Ethical clearance was obtained from the Institutional Research Committee before the commencement of the study.

## Data collection:

Data were retrieved from the donor deferral register maintained at the Blood Centre, UPUMS. This register provided comprehensive information on deferred donors, including demographic details, medical history, physical examination findings, hemoglobin levels, vital signs and risk behaviours.

## Statistical analysis:

Descriptive statistical analysis was conducted to categorize deferrals based on gender, first-time (FT) vs. repeat (RPT) donors, voluntary donors (VD) versus replacement donors (RD), and temporary vs. permanent deferrals. Donor deferral rates for various reasons were calculated as percentages. All statistical analyses were performed using SPSS software 22 (SPSS, Inc., Chicago, IL, USA).

## Results and Discussion:

A total of 45,067 participants registered to donate blood at the Blood Centre, Uttar Pradesh University of Medical Sciences (UPUMS), Saifai, Etawah, Uttar Pradesh, between March 2018 and May 2023. Among them, 43,535 (97.6%) were males and 1,532 (2.4%) were females. The overall deferral rate was found to be 18.1% (Table 1 - see PDF, Figure 1 - see PDF). A significant gender disparity was observed in deferral rates. While 17.2% of males were deferred, the deferral rate among females was much higher at 43.1%. Despite the lower deferral rate among males, they accounted for 91.9% of total deferrals, while females represented 8.1% of the total deferred donors. Figure 1 (see PDF) shows type of whole blood donor deferred showed that the majority were temporarily differed both for male and female blood donors. Only 24% of males deferred due to permanent cause and only 0.9%of female blood donors deferred due to permanent reasons. The majority of deferred donors were young adults (Table 2 - see PDF, Figure 2 - see PDF). The age distribution of deferred donors showed that the majority were younger, with 74.4% (6078 individuals) falling within the 18- to 30-year age group. Another 23.4% (1917 individuals) were aged between 31 and 45 years, and 2% (164 individuals) were aged between 46 and 60 years. Most deferrals were temporary (75%), with males comprising 81.7% and females 87.8% of this category. Permanent deferrals (25%) were slightly more common among males (18.7%) than females (12.2%) (Table 3 - see PDF). The most common temporary deferral cause was low hemoglobin (32% in males, 40% in females), followed by medication use (17.2% in males, 21% in females). Menstruation (21%) was a female-specific cause (Table 4 - see PDF). The leading permanent deferral cause was uncontrolled hypertension (36% in males, 18% in females), followed by uncontrolled diabetes mellitus (2.8% in males, 11% in females). Epilepsy (1.3% in males, 5% in females) was the least common cause (Table 5 - see PDF). Overall, permanent deferrals constituted 23.6% of all deferrals. Hypertension and diabetes were major causes, indicating the importance of pre-screening strategies to reduce deferrals.

## Summary:

[1] Deferral Rate: 18.1% overall (higher in females: 43.1% vs. males: 17.2%).

[2] Temporary Deferrals (75%): Mainly due to low hemoglobin, recent medication, and menstruation.

[3] Permanent Deferrals (25%): Mostly due to hypertension, diabetes, and high-risk behaviour.

[4] Younger donors (18-30 years) constituted the majority of deferred cases (74.4%).

Assessment of blood donor deferral in a specific demographic area provides valuable insights for regional policymaking and the formulation of national blood donation policies [3]. In this study, the overall blood donor deferral rate was 18.1%, which aligns with findings from similar studies conducted in India. For example, Mangwana *et al.* reported a deferral rate of 17.88% in a tertiary healthcare centre in North India [4]. Previous studies from Western India have reported deferral rates ranging from 11% to 33%, while Srivastava *et al.* found a deferral rate of 11.5%, with most deferrals being temporary [6]. However, our findings differ from those of Agnihotri *et al.*, Sundar *et al.*, Gaajre *et al.* and Taneja *et al.* who reported lower deferral rates in urban settings [5, 7, 9, 11- 12]. This discrepancy may be attributed to higher awareness levels and better health conditions in urban populations compared to rural donors. Conversely, Shah R *et al.* reported a deferral rate as high as 33% in Western India, while studies from Southern India have documented significantly lower deferral rates, around 5% [8, 10]. Gender-wise, female donors had a higher deferral rate (43.1%) compared to males (17.2%), which aligns with studies by Sundar *et al.* who identified low hemoglobin, low body weight, and hypotension as the three most common causes of deferral among females. Among males, the leading reasons for deferral were hypertension, low body weight, and anemia [7].

## Demographic trends and deferral reasons:

The 18-30-year age group accounted for 74.4% of all deferrals, consistent with findings by Singh *et al.* and Bahadur *et al.* While young adults are often the most eligible and available blood donors, they also face a higher risk of temporary deferrals due to anemia, underweight status, or recent medication use [10, 13]. Temporary deferrals (76.4%) were significantly more common than permanent deferrals (23.6%), aligning with prior research by Custer *et al.*, Malhotra *et al.*, Belmokhtar *et al.*, Okoroiwu *et al.* and Kandasamy *et al.* [14, 15, 16, 17-18]. In this study, anemia emerged as the leading cause of temporary deferral (73.7%), followed by alcohol consumption (11.9%) (Table 4 - see PDF). These findings mirror multiple studies where anemia remains the predominant reason for deferral among both male and female donors.

## Implications for blood donation strategies:

Our findings emphasize the strong association between gender, age, and deferral type, highlighting the need for targeted interventions to address key demographic challenges. Educational campaigns focusing on dietary habits, iron supplementation, and lifestyle modifications could help reduce temporary deferrals. Regular health screenings and pre-donation counselling can further improve donor retention and minimize unnecessary deferrals. Social behaviours such as alcohol consumption, drug use, and travel history should also be considered in donor eligibility assessments to ensure blood safety. By incorporating behavioural and medical assessments, blood banks can effectively manage deferral risks while promoting a safe and sustainable blood supply.

## Limitations:

## This study has several limitations:

[1] As a retrospective study, it relies on previously recorded data, which may be incomplete or inconsistent, affecting accuracy.

[2] Conducted at a single tertiary care hospital, the findings may not be generalizable to other regions or populations.

[3] The lower number of female donors may not fully capture gender-specific deferral patterns.

[4] The study lacks follow-up data on deferred donors to determine if they later became eligible.

[5] The study period (March 2018 - May 2023) may not fully reflect trends beyond this timeframe.

[6] Sociocultural factors influencing donor deferrals, such as religious beliefs, cultural taboos, and gender norms, were not extensively explored.

## Conclusion:

Despite a moderate deferral rate of 18.1%, anemia, alcohol use, and sociocultural beliefs significantly influence blood donation trends. Social and gender norms, along with misinformation about donation, remain major barriers to donor participation. Addressing these issues through targeted education, youth engagement, and supportive donor policies can reduce deferrals and ensure a safe, sustainable blood supply.

## Ethical considerations:

Ethical approval taken from the institutional ethical committee.

## Funding Statement:

Nil
